# Budget impact analysis of robot-assisted versus conventional spine surgery in Spain

**DOI:** 10.1007/s11701-026-03315-7

**Published:** 2026-05-04

**Authors:** Rubén Martín Láez, Fernando Álvarez-Sala Walther, Julen Monje, María Álvarez, Mehdi Zahra, Sofía De Pedro, Paula Castro Albarrán, Miguel Ángel Casado, José A. Fernández Alén

**Affiliations:** 1 Department of Neurosurgery & Surgical Spine Unit, Marqués de Valdecilla University Hospital, Cantabria, Spain; 2 Spinal Pathology Unit, Ruber International Hospital, Madrid, Spain; 3 Medtronic Ibérica, S.A., Madrid, Spain; 4 Medtronic International Trading Sàrl, Tolochenaz, Switzerland; 5 Pharmacoeconomics & Outcomes Research Iberia (PORIB), Madrid, Spain; 6 Department of Neurosurgery, La Princesa University Hospital, Madrid, Spain; 7 Universidad Autónoma de Madrid, Madrid, Spain

**Keywords:** Robot-assisted spine surgery, Budget impact analysis, Return on Investment, Spain

## Abstract

Robot-assisted spine surgery (RASS) enhances procedural accuracy and reproducibility by ensuring full adherence to preoperative surgical plans. However, its widespread adoption remains limited by the need for a substantial upfront investment. In this study, the economic impact of spinal procedures performed under the guidance of the Mazor™ robotic system was compared with that of conventional techniques from an advanced Spanish hospital perspective. A 10-year budget impact analysis was conducted. The target population included all spinal surgeries potentially suitable for robot assistance, estimated from published data and expert input. Two scenarios were compared: a baseline scenario without Mazor™ and a progressive Mazor™ adoption scenario. Unit costs (€, 2025), obtained from Spanish sources, were applied to components of health care resource consumption. Model inputs and assumptions were validated by a panel of Spanish experts. A deterministic one-way sensitivity analysis (OWSA) was performed to assess the robustness of the results. The number of RASS-eligible patients increased from 213 to 330 annually over 10 years (2,673 total). The mean cost per patient was €10,093 for conventional surgery and €9,082 for RASS, generating average savings of €1,011 per patient. Savings were driven mainly by reductions in the length of hospital stay (€1,523), revision surgeries (€793), and complications (€261), outweighing the Mazor™ acquisition cost. Over the 10-year horizon, cumulative savings reached €2.54 million, achieving full capital investment recovery within 3.83 years. OWSA confirmed the robustness of these findings. From an advanced Spanish hospital perspective, the initial investment in RASS is recouped within four years, supporting its financial sustainability and long-term economic advantage over conventional spinal surgery.

## Introduction

Spine surgeries have been increasingly performed over the past decades, in large part because of the increasing prevalence of osteoporosis and degenerative spine diseases among the aging population [1]. Globally, the annual number of spine surgeries has increased by 1.9–2.4 times over the past years [2–4]; specifically, in Europe, the number of spine surgeries is predicted to increase, with a compound annual growth rate of 3.5% between 2019 and 2030 [5].

As a consequence, procedures aimed at spinal stabilization, such as lumbar and thoracolumbar fusions are now routinely performed [6, 7]. These surgeries typically involve pedicle screw insertion, which are technically demanding because of the complex vertebral anatomy and proximity to critical neurovascular structures [6, 8]. Consequently, accuracy is essential for preventing complications such as spinal cord or nerve root injury [6, 8]. In this context, traditional freehand and fluoroscopy-guided techniques face several limitations, including variations in screw placement accuracy [8], inconsistency between surgical plans and execution [9], limited tools for comprehensive construct planning [10], and ongoing concerns regarding radiation exposure during intraoperative imaging [7].

The need to improve surgical outcomes and minimize surgery-related risks has prompted significant technological innovations to circumvent the limitations of conventional techniques [11]. These methods range from the use of visualization systems, such as computed tomography-guided navigation, to robotic guidance systems such as Mazor™, which not only increase mechanical stability during pedicle screw insertion but also support preoperative modeling and holistic visualization [12, 13].

Several studies have reported that robot-assisted spine surgery (RASS) may increase screw placement accuracy [14, 15], reduce facet joint violations [16], decrease the rate of postoperative complications (especially revision surgery due to pedicle screw malposition) [17, 18], decrease the hospital length of stay (LOS) [19] and decrease radiation exposure [20]. Furthermore, considering that robotic systems decrease the invasiveness of surgical approaches, their integration has the potential to improve clinical outcomes and reduce blood loss, postoperative pain, and recovery times [15, 21–23], ultimately leading to improved patient-reported experiences and outcomes (PRE/PRO) [24].

Despite all these potential clinical benefits, the widespread adoption of RASS remains constrained by significant upfront investment costs, which may be perceived as a key barrier for many hospitals [25]. However, RASS may reduce overall hospital resource utilization, potentially offsetting part of their high investment costs by shortening the LOS or reducing the need for other resource-intensive interventions. Consequently, the integration of robotic technology has the potential to partially balance the upfront expenses through improved efficiency and reduced short/medium-term costs [26]. However, the exact extent of this impact remains largely unknown.

In settings where public resources are limited, evaluating new technologies from an economic perspective is important. RASS is a key example, as it may represent a paradigm shift with broad implications for health care systems, not only for day-to-day practice but also in terms of sustainability. Consequently, in this study, the budget impact of implementing the Mazor™ robotic system in spinal surgery was compared with that of conventional techniques from the perspective of an advanced Spanish hospital.

## Methods

### Model overview

A cohort-based budget impact analysis (BIA) was developed in Microsoft Excel^®^ 2024 (Microsoft Corporation, Redmond, WA, USA) to estimate the economic consequences of integrating, into an advanced Spanish hospital, the Mazor™ robotic system into spine surgeries over a 10-year time horizon. Two scenarios were compared: (1) a baseline scenario in which all eligible patients underwent conventional spine surgery (freehand or fluoroscopy-guided), and (2) an alternative scenario in which one Mazor™ system was progressively adopted for all suitable RASS. Each year, a new cohort of eligible patients was evaluated independently. The model estimated both annual and cumulative direct health care costs, including the acquisition and operational costs of the robotic system, surgical resource use, LOS, and postoperative complications.

The primary outcomes were the return of investment (ROI) associated with adopting the Mazor™ system, calculated as the number of years required to offset the initial capital investment through per-patient cost savings, and the budget impact, defined as the difference in total costs between the two evaluated scenarios. A 10-year time frame was chosen to reflect typical amortization cycles and to capture long-term trends in technology adoption.

Results are presented as cost per patient and cost per cohort. Resource use and complications are assumed to be linear over the entire time horizon. However, total costs vary by year due to the phased adoption of RASS and the expected increase in the number of incident cases each year.

### Clinical expert panel

To ensure that the model accurately reflected clinical practice and health care resource use in Spain, a panel of three experienced surgeons was convened. The panel included two neurosurgeons and one orthopedic surgeon, each with substantial experience in spinal surgery and representing different geographic regions and hospital types (both public and private). This diverse panel was created to capture national variability in clinical workflows and patient profiles.

Model parameters requiring expert validation included patient eligibility for robot assistance, adoption rates, surgical team composition, revision and complication rates, and standard care pathways. A structured questionnaire was developed to collect individual expert input on each parameter. The experts completed the questionnaire independently, after which a face-to-face consensus meeting was held to discuss discrepancies and agree on the final values. In the absence of local data, consensus values were used as primary inputs; where applicable, expert estimates were triangulated with published literature.

The expert panel also provided input for interpreting uncertain parameters and establishing plausible upper and lower bounds for use in sensitivity analyses (SAs).

### Literature review

A comprehensive literature review of articles published in English between 1990 and 2025 was conducted. The MEDLINE database via PubMed was used to identify scientific publications related to conventional surgery and RASS, with the objective of informing model conceptualization, identifying relevant parameters, and extracting data for the analysis. The search terms combined Medical Subject Headings (MeSH) and free-text keywords, including “robot-assisted”, “robotic guidance”, “spine surgery”, “pedicle screw”, “conventional surgery”, “fluoroscopy-guided”, and “freehand”, and Boolean operators (AND/OR) were used.

Studies were critically screened and appraised according to predefined criteria, and those with high levels of evidence were selected. The type of study was considered. Case series and case reports, operative videos, cadaver studies, narrative reviews and letters to the author were excluded. Multicenter studies were preferred over single-center studies. Second, the methodological quality of the studies was evaluated, including randomization, anonymization, sample size, inclusion/exclusion criteria and robustness of the statistical analyses. These also applied to the studies included in the systematic literature review and meta-analysis. Third, recent studies conducted in or with the participation of Spain (or, failing this, Europe) were preferred. Fourth, comparators were also considered, and articles that directly compared conventional techniques with RASS were selected. Last, articles with values directly from original sources were preferred; otherwise, the sources and methodology used to obtain the values described should be noted.

### Population

The target population consisted of patients eligible for RASS in an advanced Spanish hospital. For the purposes of this analysis, an “advanced hospital” was defined as a high-complexity center functioning as a referral institution and equipped to adopt advanced technologies such as robotic systems [27].

On the basis of clinical expert input and internal hospital volumes, the annual number of spine procedures performed at such a hospital type was estimated to be 425 during the first year of the time horizon. To account for the natural increase in surgical demand over time, and based on both, the published literature [1, 4] and expert panel judgment, a yearly growth rate of 5% was applied to reflect the Spanish healthcare context.

In the base case, for both scenarios, it was assumed that 50% of all spinal surgeries performed annually would be eligible for robot assistance. This estimate, which is based on expert consensus, reflects the proportion of procedures from the typical case mix that could be reasonably appropriate for robot guidance (e.g., instrumented fusion or deformity correction). This eligibility assumption remained constant over the 10-year time horizon.

**Fig. 1:**
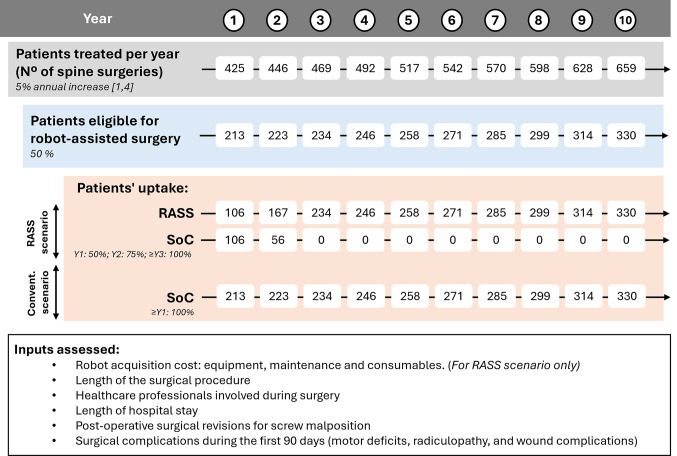
Target population and scenarios assessed per year. RASS, Robot-assisted spine surgery; SoC, Standard of Care; Y, Year. The number of RASSs (x_n_) per year was calculated using the following formula: X_n_$\;=n_x \times z \times i$, where nₓ is the total number of spine surgeries per year, z is the percentage of patients eligible for RASS, and i is the percentage of eligible surgeries performed with a robot. The number of SoC surgeries (y_n_) per year was calculated using the following formula: y_n_ = *n*_*x*_× *z* - X_n_

The population flow and annual number of surgeries performed over the time horizon are illustrated in Fig. 1.

### Scenarios assessed

A BIA was performed to compare two different scenarios: (1) the conventional surgery scenario and (2) the RASS scenario.

In the conventional surgery scenario, all eligible patients underwent conventional surgery during the 10-year time horizon. Conversely, in the RASS scenario, for all suitable RASS (50%), a gradual adoption of the Mazor™ system was modeled over the time horizon to reflect real-world implementation dynamics, which was informed by clinical experience with similar technologies in Spain. 50% and 75% of surgeries that were eligible for RASS were performed with Mazor™ in years 1 and 2, respectively, with full adoption (100%) from year 3 onward. This staged approach mirrors the implementation patterns of new technologies in Spanish hospitals. A schematic representation of both scenarios is provided in Fig. 1.

### Resource consumption and cost

In line with the chosen perspective, only direct health care costs associated with spine surgeries were considered. These included resources used during the index procedure, as well as costs related to postoperative revision surgeries and complications occurring within 90 days of the index surgery.

The key resource categories incorporated in the model were acquisition costs of the robotic system (including capital expenditure, annual maintenance, consumables and training of personnel), intraoperative costs (cost of standard operating room and personnel, including surgeons, anesthesiologists, nurses, and technical assistants); hospital LOS; postoperative revision surgeries due to screw misplacement; and surgical complications, including motor deficits, radiculopathy, and wound complications, categorized by severity and treatment type (e.g., conservative management vs. decompression or laminectomy). To avoid double counting, fusion procedures performed for the management of complications were excluded from the complication category when they overlapped with the definition of revision surgery (i.e., screw repositioning). Moreover, no costs associated with hospital infrastructure changes were included in the analysis, as the robotic system acquisition does not require any structural modifications or changes to existing hospital equipment.

Resource consumption values for each scenario (conventional vs. RASS) were obtained from the published literature [15, 21, 28] and validated by the expert panel. When no data were available or when existing data did not reflect the national context, expert-derived estimates were applied in line with the available evidence, as was the case for revision surgeries, for which assumptions were informed by data on the use of navigation in spine surgery [29].

All unit costs were obtained from publicly available Spanish sources [30, 31], including a national health database that collects health costs from different sources and official remuneration tables from autonomous communities. Costs were updated to 2025 euros (€) using appropriate inflation adjustments when necessary. Only the acquisition cost of the robotic system was specific to Mazor™. The analysis did not account for indirect costs (e.g., productivity loss, long-term disability), as these are outside the scope of a hospital budget perspective.

The full list of resource use parameters, unitary costs and sources used in the model is detailed in Table 1.

**Table 1:** Resource consumption per surgery and unit cost

Parameter	Conventional surgeries	RASS	Unit Cost (€,2025)	Reference
Length of surgery*	196.8 min	203.5 min	€ 522.7 per hour	• Parameter: Siccoli et al. 2019 [21] • Cost: Rodríguez et al. 2019 [30]
Specialists per surgery	2	2	€ 28.4 per hour	• Parameter: Experts’ panel • Cost: Average remuneration tables of the Autonomous Communities
Anesthesiologists per surgery	1	1	€ 28.4 per hour	
Nurses per surgery	2	2	€ 17.7 per hour	
Technical assistants per surgery	1	1	€ 12.4 per hour	
Length of hospital stay*	5.35 days	4.15 days	€ 1,269.4 per day	• Parameter: Tovar et al. 2022 [15] • Cost: e-Salud [31]
Postoperative revision**	5.0%	0.5%	€ 17,630.0 per surgery**	• Parameter: Experts’ panel (based on [29]) • Cost: e-Salud and CMBD [31, 45]
**Complications (90 days)**				
Motor deficit	• Conservative management: 1.80% • Decompression/Laminectomy: 0.90%	• Conservative management: 0.30% • Decompression/Laminectomy: 0.00%	• Conservative management: € 214.0*** • Decompression/Laminectomy: € 6,442.2	• Parameter: Liounakos et al. 2021 [28] • Cost: e-Salud [31]
Radiculopathy	• Conservative management: 13.50% • Decompression/Laminectomy: 0.90%	• Conservative management: 2.60% • Decompression/Laminectomy: 0.00%		
Wound complications	• Conservative management: 5.40% • Deep infection: 1.80%	• Conservative management: 1.10% • Deep infection: 0.00%	• Conservative management: € 214.0*** • Deep infection: € 6,089.2	

RASS: Robot-assisted spine surgery

*The durations in the conventional scenario were calculated by multiplying the average time reported in each of the articles included in the meta-analyses by its assigned weight (in %). To determine the duration of robotic surgery, the difference in time reported in the meta-analyses was applied to the conventional surgery time obtained previously. **The cost of postoperative revision surgeries was estimated using diagnostic-related groups (DRGs) associated with minor severity dorsal and lumbar fusion procedures, differentiating “dorsal and lumbar fusion procedures except for scoliosis” from “dorsal and lumbar fusion procedures for scoliosis”. For each, the number of procedures obtained from the CMBD [45] and their associated cost obtained from the average unit costs extracted from the e-Salud database [31] were considered. In the case of dorsal and lumbar fusion procedures except for scoliosis (minor severity), the number of contacts in 2023 was 19,210, with an average cost of €16,878.5. In the case of dorsal and lumbar fusion procedures for scoliosis (minor severity), the number of contacts in 2023 was 1,203, with an average cost of €29,679.0. The average cost for postoperative revision surgery was then estimated. *** Assumed to be an outpatient neurosurgery consultation

### Sensitivity analysis

To assess the robustness of the base-case results and account for parameter uncertainty, a deterministic one-way sensitivity analysis (OWSA) was conducted. Each input parameter was varied individually within a plausible range, while all other parameters were kept constant, allowing assessment of its isolated impact on the model results, including both per-patient cost differences and the ROI.

The following parameters were tested in the OWSA:

- Difference in LOS between RASS and conventional techniques (− 1.69 days; −0.62 days) [15].
- Time horizon (5 years).
- Annual number of spinal surgeries (250; 600).
- Unit costs (± 20%).
- Progressive increase in the proportion of procedures eligible for robot assistance over the time horizon (50% to 80%).
- Difference in operative time between RASS and conventional techniques (24.3 min; 0 min) [15].
- Discount rate (3%; 5%).
- Difference in the revision surgery rate due to malposition between RASS and conventional techniques (± 20%).
- Annual growth in the number of spinal surgeries (3.5%; 6.5%) [1, 4].
- Difference in the complication rate between RASS and conventional techniques (± 20%).
- Robot-assistance adoption rate by year (Year 1: 50%; Year 2: 75%; Year 3: 85%; Year 4 and beyond: 100%).

Variation ranges were derived from published literature, where available, or determined through expert consensus when empirical data were lacking. The results were graphically presented using a tornado diagram to highlight the most influential parameters on both cost outcomes and time to ROI.

## Results

### Base case results

Over the 10-year time horizon, the number of patients eligible for RASS in an advanced Spanish hospital is projected to increase from 213 in year 1 to 330 in year 10, resulting in a cumulative cohort of 2,673 patients.

RASS was associated with a 3.2% increase in operative time and surgical staff hours. Notably, RASS also led to a 21.1% reduction in the length of hospital stay, a 78.5% reduction in surgery-related complications, and an 84.5% reduction in postoperative revision surgeries.

**Fig. 2:**
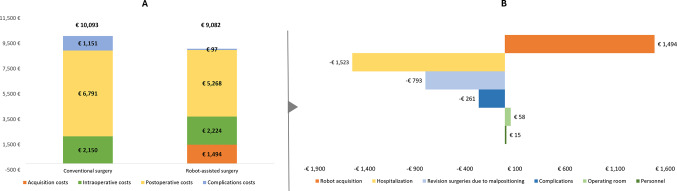
Costs per surgery. (**A**) Cost per type of surgery. (**B**) Differences in disaggregated costs per surgery

The mean cost per patient was estimated to be €10,093 for conventional surgery and €9,082 for RASS, yielding an average cost savings of €1,011 per patient (Fig. 2A). While RASS incurred higher per-patient costs due to acquisition (€1,494) and intraoperative resources (€73), these costs were offset by savings from a reduced length of hospital stay (–€1,523), fewer revision procedures (–€793), and lower costs for the management of surgery-related complications (–€261) (Fig. 2B).

**Fig. 3:**
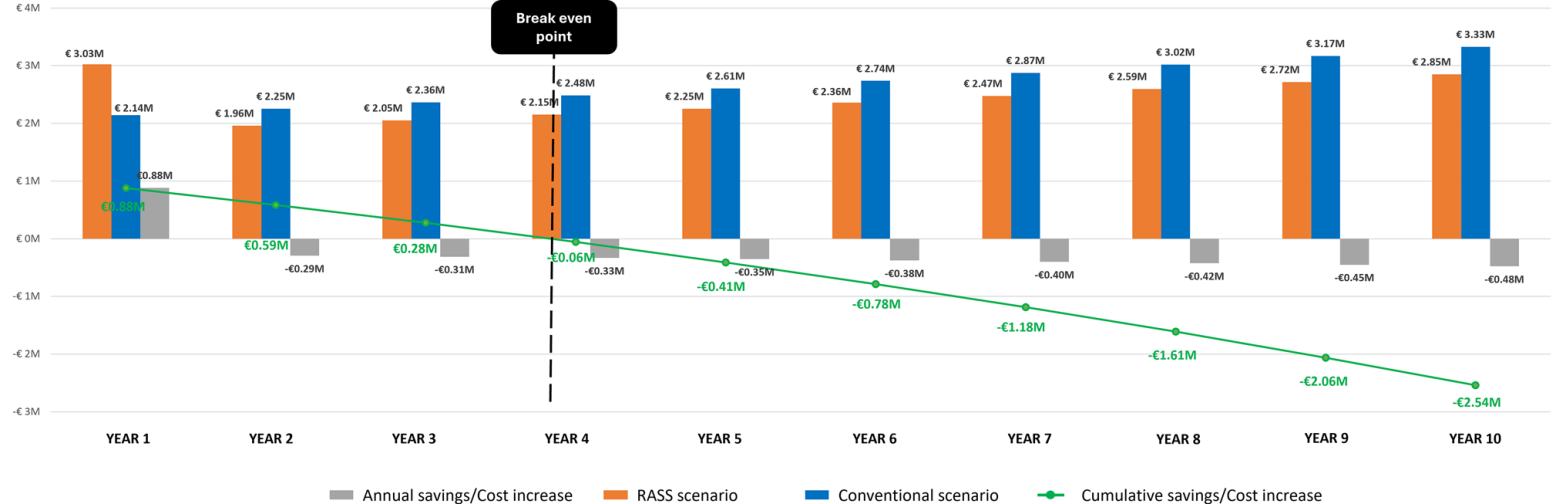
ROI estimation times for the overall target population. RASS: Robot-assisted spine surgery; ROI: return of investment

At the population level, the total costs at 10 years were €26,976,748 in the conventional scenario versus €24,437,993 in the RASS scenario, generating net budget savings of €2,538,755 (Table 2). The initial capital investment associated with the Mazor™ system fully recovered within 3.83 years, as illustrated in Fig. 3, with a total of 712 surgeries performed.

**Table 2:** Budget impact analysis for the overall target population

	Year	1	2	3	4	5	6	7	8	9	10	TOTAL
Acquisition	Conventional	€0	€0	€0	€0	€0	€0	€0	€0	€0	€0	€0
	RASS	€1,146,688	€125,508	€275,711	€284,496	€293,721	€303,407	€313,578	€324,257	€335,469	€347,243	€3,750,078
	Difference	€1,146,688	€125,508	€275,711	€284,496	€293,721	€303,407	€313,578	€324,257	€335,469	€347,243	€3,750,078
Operation room	Conventional	€364,336	€382,553	€401,680	€421,764	€442,852	€464,995	€488,245	€512,657	€538,290	€565,204	€4,582,577
	RASS	€370,538	€392,321	€415,355	€436,123	€457,929	€480,826	€504,867	€530,110	€556,616	€584,447	€4,729,132
	Difference	€6,202	€9,768	€13,675	€14,359	€15,077	€15,831	€16,622	€17,453	€18,326	€19,242	€146,555
Health care professionals	Conventional	€92,612	€97,243	€102,105	€107,210	€112,571	€118,199	€124,109	€130,315	€136,830	€143,672	€1,164,867
	RASS	€94,189	€99,726	€105,581	€110,860	€116,403	€122,223	€128,335	€134,751	€141,489	€148,563	€1,202,120
	Difference	€1,576	€2,483	€3,476	€3,650	€3,832	€4,024	€4,225	€4,437	€4,658	€4,891	€37,253
Hospitalization	Conventional	€1,443,161	€1,515,319	€1,591,085	€1,670,639	€1,754,171	€1,841,880	€1,933,974	€2,030,673	€2,132,206	€2,238,816	€18,151,925
	RASS	€1,281,311	€1,260,406	€1,234,206	€1,295,916	€1,360,712	€1,428,748	€1,500,185	€1,575,195	€1,653,954	€1,736,652	€14,327,286
	Difference	-€161,850	-€254,913	-€356,879	-€374,723	-€393,459	-€413,132	-€433,789	-€455,478	-€478,252	-€502,164	-€3,824,639
Postoperative revision	Conventional	€187,319	€196,685	€206,519	€216,845	€227,687	€239,071	€251,025	€263,576	€276,755	€290,593	€2,356,075
	RASS	€103,025	€63,923	€20,652	€21,684	€22,769	€23,907	€25,103	€26,358	€27,676	€29,059	€364,155
	Difference	-€84,293	-€132,762	-€185,867	-€195,160	-€204,918	-€215,164	-€225,923	-€237,219	-€249,080	-€261,534	-€1,991,920
Complications	Conventional	€57,347	€60,214	€63,225	€66,386	€69,706	€73,191	€76,851	€80,693	€84,728	€88,964	€721,305
	RASS	€29,583	€16,486	€2,006	€2,106	€2,211	€2,322	€2,438	€2,560	€2,688	€2,822	€65,222
	Difference	-€27,764	-€43,728	-€61,219	-€64,280	-€67,494	-€70,869	-€74,413	-€78,133	-€82,040	-€86,142	-€656,083
Total	Conventional	**€2**,**144**,**775**	**€2**,**252**,**014**	**€2**,**364**,**614**	**€2**,**482**,**845**	**€2**,**606**,**987**	**€2**,**737**,**337**	**€2**,**874**,**204**	**€3**,**017**,**914**	**€3**,**168**,**809**	**€3**,**327**,**250**	**€26**,**976**,**748**
	RASS	**€3**,**025**,**334**	**€1**,**958**,**369**	**€2**,**053**,**511**	**€2**,**151**,**187**	**€2**,**253**,**746**	**€2**,**361**,**433**	**€2**,**474**,**505**	**€2**,**593**,**230**	**€2**,**717**,**892**	**€2**,**848**,**786**	**€24**,**437**,**993**
	Difference	**€880**,**559**	**-€293**,**645**	**-€311**,**103**	**-€331**,**658**	**-€353**,**241**	**-€375**,**903**	**-€399**,**698**	**-€424**,**683**	**-€450**,**918**	**-€478**,**463**	**-€2**,**538**,**755**

RASS, Robot-assisted spine surgeries

### Sensitivity analyses

**Fig. 4:**
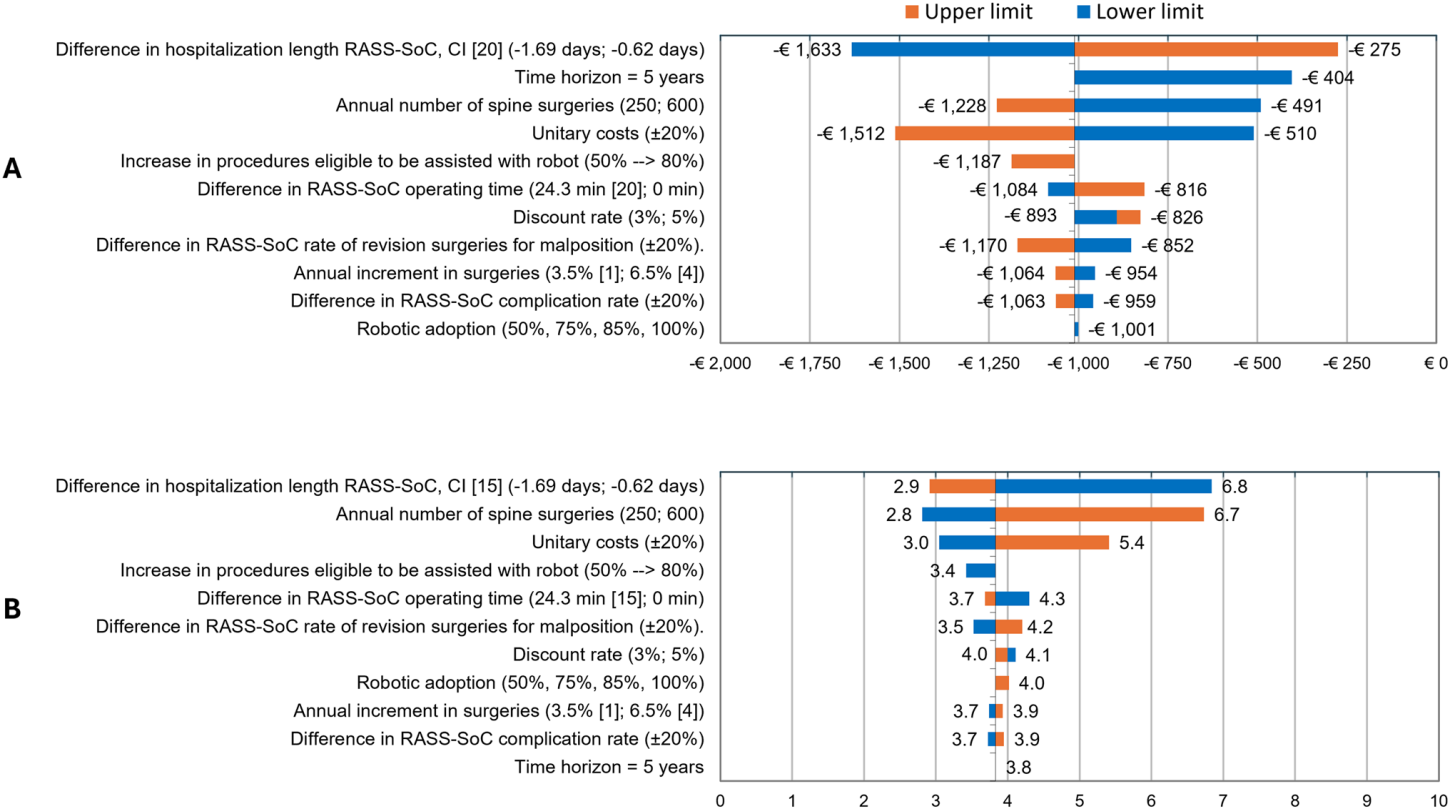
OWSA results. (**A**) Cost analysis results per patient. (**B**) ROI results for the overall target population. CI: Confidence interval; OWSA: One-way sensitivity analysis; RASS-SoC: Robot-assisted spine surgery versus Standard of Care; ROI: return of investment

OWSA confirmed the robustness of the base-case results. Across all tested parameters, the per-patient cost savings associated with RASS ranged from €275 to €1,633. The variables that had the greatest influence on cost savings were hospital LOS, followed by the time horizon and the number of annual spine surgeries (Fig. 4A).

The estimated time to ROI ranged from 2.8 to 6.8 years, depending on the variation in key inputs, with the length of hospital stay, annual number of surgeries and unit costs being, in that order, the parameters that most influenced the results (Fig. 4B).

## Discussion

The acceptance and integration of robotics into spine surgeries is steadily increasing, driven by a growing body of evidence that highlights its clinical advantages [14]. Nevertheless, decisions concerning the implementation of new technologies in publicly funded health care systems, such as the Spanish National Health System, should also take into account the financial perspective.

The present analysis was conducted to estimate the economic impact that the adoption of Mazor™ would have in an advanced Spanish hospital choosing to implement this technology into its spinal surgery practice. The results showed that despite the initial investment, the integration of robotics lowers the cost per procedure by €1,011, resulting in a reduced budget impact. Indeed, this analysis estimated cost savings of €2,538,755 over a 10-year time horizon for the whole target population, driven mainly by a reduction in the LOS and the number of postoperative complications. Additionally, it was estimated that the initial investment could be recovered within four years of Mazor™ incorporation into spinal surgery.

Currently, although multiple areas of health care are shifting toward robotic technologies, widespread implementation is often hindered by high upfront costs [25], particularly in publicly funded health care systems, where the broader implications for efficiency and long-term sustainability should be assessed and are often considered critical for the decision-making process. The results obtained in the present analysis suggest that RASS offers an opportunity to optimize key aspects of the surgical care pathway. These improvements not only translate into better clinical outcomes for patients but also into more efficient use of health care resources. Therefore, the implementation of robotic systems in spine surgery may represent a strategic investment that enhances the overall performance of the health system. In this context, economic evaluations such as this one are essential for supporting evidence-based decision-making, allowing proper resource allocation for innovative technologies that could improve both patient care and system efficiency in the medium and long term.

Preoperative planning plays a critical role in RASS by minimizing surgical variability associated with the multifaceted hurdles of spinal surgery [32]. Surgeons can define the optimal entry point, trajectory, and dimensions of the implants, providing consistent and reproducible precision across different operators, particularly in complex cases or anatomically challenging patients, where variability is more pronounced [12, 13, 23]. Robotic guidance also enhances predictability, allowing surgeons to anticipate anatomical challenges and make decisions prior to the procedure. Overall, this results in improvements not only in the accuracy of screw placement and reduced complication rates but also in lower radiation exposure [12].

This reduction in radiation exposure is especially relevant given the growing concern about cumulative radiation doses in operating rooms [33]. Conventional spine surgeries often rely on fluoroscopic imaging to verify implant positioning, resulting in low but persistent radiation exposure, especially for operating room staff [34]. Conversely, when robotic guidance is used, navigation is primarily based on preoperative imaging and real-time feedback, reducing the need for continuous fluoroscopy and thereby decreasing the radiation dose [12]. The side effects of continuous small doses of radiation remain unclear [34], but existing evidence tends to link it to an increased risk of certain types of cancer, lens opacity and cell damage [35].

The learning curve inherent to RASS is a critical factor in maximizing the aforementioned benefits, such that increased experience with this technology over time is likely to improve the clinical and economic outcomes achieved even further [36]. The effect of the learning curve was not explicitly evaluated in this study due to the lack of data and the complexity that considering it would have added to the model. However, different inputs such as the length of surgery and hospital stay were derived from a meta‑analysis including retrospective studies conducted during the introduction of the robotic system, thereby implicitly capturing part of the learning curve effect. It is reasonable to expect that, like computer-assisted navigation [37], as surgeons become familiar with the RASS workflow and gain experience with the use of the robotic system, an improvement in parameters related to surgical efficiency can be anticipated. Once the learning curve has been surpassed and the surgeon has become an expert, the operative time could be further reduced, and the overall accuracy could increase, expanding the long-term benefits of RASS in the same manner that has been documented for navigation [38].

Beyond the benefits described during the analysis, there are other advantages that should be considered when establishing the overall value of RASS. First, the use of robotic guidance promotes minimally invasive surgery, which is broadly associated with faster recovery, reduced intraoperative blood loss and transfusion requirements, less postoperative pain, and fewer long-term complications, such as adjacent segment disease [39]. This complication, characterized by accelerated degenerative changes at spinal levels adjacent to the fused segment, can lead to chronic pain or new neurological symptoms and ultimately may require revision surgery [40]. As facet joint violation during index surgery is a well-established predictor of adjacent segment degeneration [22], and RASS has been shown to markedly reduce its incidence [22], it may also reduce the long-term need for revision surgery.

Additionally, different studies have reported improved patient satisfaction and functional outcomes, measured using validated scales (e.g., the Scoliosis Research Society 22-Item Questionnaire, Oswestry Disability Index, and Numeric Rating Scale), after RASS procedures [41]. This aligns with the idea that better clinical outcomes and a lower risk of surgical complications improve PREs and PROs by reducing postoperative pain and recovery time. Accelerated recovery also favors a quicker return to work and therefore a reduction in productivity loss. Although data related to absenteeism are limited, a shorter hospital stay is correlated with faster mobilization and likely fewer days off work [42], which could mean even lower costs if the societal perspective is taken into account.

Nonetheless, high-quality evidence is still needed to comprehensively incorporate these aspects into an economic analysis and fully capture the value of RASS. Future research could benefit from exploring the impact of the learning curve, adopting a societal perspective to account for indirect costs associated with both RASS and conventional surgery, and investigating how improvements in PROs and PREs translate into enhanced quality of life—and consequently better health outcomes—through economic evaluations, such as cost-utility analyses.

To the authors’ knowledge, this is the first BIA to explore the economic impact of RASS in Spain. Previous studies published in other contexts have shown similar results, with a downward trend in costs, which is driven mainly by shorter hospital stays [43, 44]. The present analysis explores economic concepts beyond hospital LOS and the cost of intervention, enhancing understanding and decreasing financial uncertainty around RASS adoption.

To allow correct interpretation of this study’s results, several limitations must be acknowledged. First, a financially conservative approach was adopted because of the unclear economic impact of some factors, which, if included, might have substantially influenced the results. Facet joint violations, radiation exposure to patients and staff, fluoroscopy guidance system costs and indirect costs were not incorporated into the analysis. In the same way, complications such as pseudoarthrosis, back pain, minor urological problems, acute myocardial infarction or pulmonary embolism were also excluded because they were not considered to be related to the surgical procedure and because their incidence is unlikely to differ between conventional surgery and RASS. However, consideration of these parameters would have potentially led to results in favor of RASS, increasing the 10-year cost savings and reducing the time to ROI.

Second, when Spanish-specific data were unavailable, values from other countries or consensus from the panel of experts were selected. Third, significant variability among hospitals in terms of clinical practice, surgical approach, and caseload should be noted, as this variability may affect the number of procedures performed, clinical outcomes and costs. To overcome this limitation, the expert panel was composed of surgeons from different locations and hospital types. Additionally, whenever different costs were identified, the average cost was calculated and considered in the SA. Similarly, the assumptions reached by the experts regarding the annual number of spine surgeries, their eligibility for being RASS, and the annual growth rate of performed surgeries, were also tested in the SA.

Finally, considering the lack of evidence on resource consumption stratified by indication and type of surgery, the analysis assumed that all spinal surgeries were managed in the same way. To address this limitation, priority was given to those studies that included populations with various types of indications and procedures, as they are more likely to reflect the surgical case mix of an advanced hospital.

All these limitations could be overcome, to some extent, by incorporating national real-world evidence, both for the number of procedures and resource utilization. Thus, despite the fact that the meta-analyses considered already include real-world studies, it would be interesting to develop, in the future, studies focused on the collection of real-world data from patients undergoing spinal surgeries to further strengthen robustness and external validity of studies of this type. Nevertheless, in the present study, all the data inputs and assumptions were validated by an expert panel with extensive experience in spinal surgery. Furthermore, the results of the OWSA confirmed the robustness of the analysis, as the variation in different parameters associated with uncertainty did not result in a significant deviation from the base case results.

## Conclusion

The results of this study suggest that the adoption of RASS using the Mazor™ system can be a financially sustainable strategy for advanced Spanish hospitals. Although the implementation of robotic systems entails a substantial upfront investment, the model projected that this cost would be fully offset within four years through reductions in the length of hospital stay, surgical revisions, and postoperative complications. Over a 10-year period, RASS was associated with both per-patient and cumulative cost savings, supporting its medium/long-term economic viability. These findings highlight the potential of robotic technologies to increase surgical precision and improve resource efficiency within hospital-based health care systems.

## Data Availability

All data supporting the findings of this study are available within the manuscript.
